# Evaluation of docking procedures reliability in affitins-partners interactions

**DOI:** 10.3389/fchem.2022.1074249

**Published:** 2022-12-01

**Authors:** Anna Ranaudo, Ugo Cosentino, Claudio Greco, Giorgio Moro, Alessandro Bonardi, Alessandro Maiocchi, Elisabetta Moroni

**Affiliations:** ^1^ Department of Earth and Environmental Sciences, University of Milano-Bicocca, Milan, Italy; ^2^ Department of Biotechnology and Biosciences, University of Milano-Bicocca, Milan, Italy; ^3^ Bracco SpA, Milan, Italy; ^4^ Institute of Chemical Sciences and Technologies “G. Natta”, National Research Council of Italy, SCITEC-CNR, Milan, Italy

**Keywords:** protein-protein interaction, molecular docking, affitins, DockQ, matrix of local coupling energies, consensus approach, diagnostic probes, antibody mimetics

## Abstract

Affitins constitute a class of small proteins belonging to Sul7d family, which, in microorganisms such as *Sulfolobus acidocaldarius*, bind DNA preventing its denaturation. Thanks to their stability and small size (60–66 residues in length) they have been considered as ideal candidates for engineering and have been used for more than 10 years now, for different applications. The individuation of a mutant able to recognize a specific target does not imply the knowledge of the binding geometry between the two proteins. However, its identification is of undoubted importance but not always experimentally accessible. For this reason, computational approaches such as protein-protein docking can be helpful for an initial structural characterization of the complex. This method, which produces tens of putative binding geometries ordered according to a binding score, needs to be followed by a further reranking procedure for finding the most plausible one. In the present paper, we use the server ClusPro for generating docking models of affitins with different protein partners whose experimental structures are available in the Protein Data Bank. Then, we apply two protocols for reranking the docking models. The first one investigates their stability by means of Molecular Dynamics simulations; the second one, instead, compares the docking models with the interacting residues predicted by the Matrix of Local Coupling Energies method. Results show that the more efficient way to deal with the reranking problem is to consider the information given by the two protocols together, i.e. employing a consensus approach.

## Introduction

**FIGURE 1:**
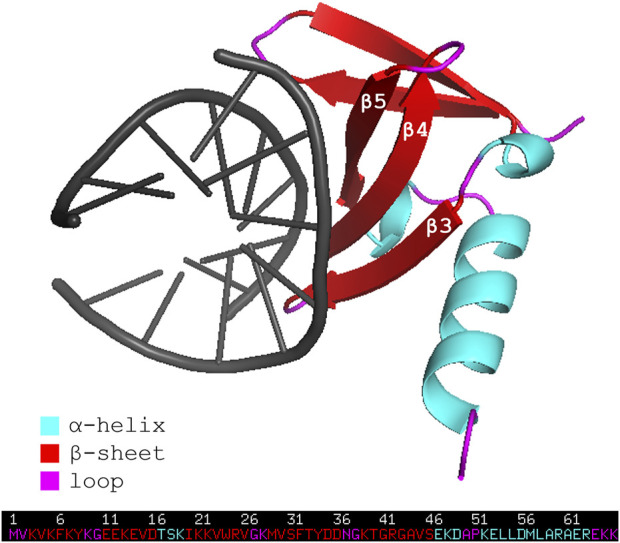
Representation of the wild-type affitin Sac7d bound to DNA duplex d(GTAATTTAC)_2_ (PDB code: 1AZQ). Affitin residues are colored following the secondary structure assignment, as shown in the legend. DNA is shown in black.

Affitins are 7-kDa proteins engineered from the naturally occurring DNA-binding protein family termed Sul7d (Kalichuk et al., 2016). Proteins of this family, such as Sac7d and Sso7d, are expressed respectively by extremophile organisms *Sulfolobus acidocaldarius* and *Sulfolobus solfataricus*, and act to prevent DNA denaturation thanks to their stability in a broad range of temperature (up to 100°C) and pH (from 0 up to 12). The general topology of Sac7d is that of the OB-fold family. Its tertiary structure consists of a five-stranded incomplete *ß*-barrel (β1 = residues 3-8, β2 = 11–16, β3 = 20–26, β4 = 29–36, β5 = 39–46), capped at the opening by a three-turn C-terminal α-helix (residues 53–63). The triple-stranded *ß*-sheet (β3-β4-β5) has been identified as the DNA binding surface (Figure 1) (Robinson et al., 1998).

Notably, this region can be engineered in order to obtain a binding specificity also towards protein targets (Mouratou et al., 2007; Krehenbrink et al., 2008; Correa et al., 2014; Béhar et al., 2016; Goux et al., 2017; Kauke et al., 2017; Mukherjee et al., 2020; Zajc et al., 2020).

Modulating the binding affinity of affitins towards specific targets can be particularly useful in the perspective of employing affitins as antibody mimetics. Briefly, antibody mimetics are alternative to the antibodies and have been conceptualized with the idea of overcoming the drawbacks that the latter present, such as low stabilities, resulting in a more complicate production, slow and inefficient tissues penetration and slow clearance from the body (Vazquez-Lombardi et al., 2015).

Affitins, being small and highly stable proteins, have thus been considered as potential antibody mimetics. In fact, their small dimensions favor an efficient tissue penetration and their high stability make them ideal scaffolds for engineering and easy to be produced.

Among the examples found in literature, the work by Goux et al. reports on the development of a molecular probe for targeting the Epidermal Growth-Factor Receptor (EGFR). This probe is constituted of an affitin (Nanofitin B10) which has been engineered in such a way to recognize the pharmacological target epidermal growth-factor receptor (EGFR) and to exhibit a unique cysteine moiety at its C-terminus. The latter is used for a fast and site-specific radiolabeling procedure necessary for the Positron Emission Tomography (PET) (Goux et al., 2017).

Affitins engineering is usually accomplished by generating large libraries of mutants, which are subsequently screened for their affinity for the selected protein target. However, once the desired mutant is obtained, no structural information about the affitin-protein complex is readily available. In such a contest, as far as computational approaches are concerned, molecular docking can be considered for an initial structural characterization.

To date, no docking program can always identify a correct solution, especially in the context of the protein-protein interactions; instead, docking results consist of a ranking of the best putative binding poses, according to the docking-score function. These require a post-docking processing, in the attempt of identifying the correct binding geometry through the analysis, scoring and, if need be, reranking of the docking models. The post-docking step can be based on energy-, knowledge-, consensus-, and evolution-based algorithms and includes, for example, the contact analysis, the prediction of the interface, the flexible refinement of the model and the energy minimization of the latter (Vangone et al., 2017).

In the perspective of using affitins as targeting scaffolds, our work focuses on the validation of the docking poses obtained for different affitin-partner complexes available in the Protein Data Bank (PDB) through two procedures described hereafter. The aim is to develop a robust protocol for the initial prediction of the most probable binding geometry between a specific target and a new engineered affitin.

Two post-docking strategies are considered in the present paper: the first one involves the investigation of the stability of docked complexes through Molecular Dynamics (MD) simulations and evaluation of CAPRI, DockQ, and other structural and energetic parameters (Jandova et al., 2021); the second one is the cross-checking between the docking models and the interaction sites predicted through the Matrix of Local Coupling Energies (MLCE) method, based on the individuation of low-intensity energetic interaction networks in the isolated protein structure (Scarabelli et al., 2010).

This paper is organized as follows. The first part presents the results of docking between seven affitins with as many protein partners whose binding experimental structures are available in the PDB. The reliability of the ranking of the docking poses is then checked by means of MD simulations and MLCE approach. Finally, we show how the coupled use of such two methods can improve the identification of the correct docking models.

## Methods

### Selection and preparation of protein structures

Protein structures were selected by doing a sequence similarity (threshold 60%) search in PDB, starting from the sequence of the wild-type affitin (PDB code: 1AZQ), which resulted in 55 entries. For our study, we selected the seven complexes made up by engineered affitins and protein partners. When available, structures of the proteins partners in the complexes were superimposed to the respective unbound forms, to check whether a significant change in their fold occurred upon binding. As this is not the case, we hold that bound structures of these proteins can be used for rigid docking calculations. All protein structures retrieved from the PDB were prepared using the tool Protein Preparation Wizard included in the Schrödinger package (Sastry et al., 2013; Schrödinger Release 2022-2, 2021). The protocol consists of: 1) elimination of water molecules and counterions, if present; 2) addition of hydrogens; 3) rebuilding of possibly missing side chains and loops with Prime (Jacobson et al., 2002, 2004); 4) optimization of hydrogen bonding network, using PROPKA for the assignment of protonation states at neutral pH; 5) minimization of hydrogen atoms.

### Affitins—Partners docking

Protein-protein docking calculations have been carried out using the web server ClusPro (Kozakov et al., 2013, 2017; Vajda et al., 2017; Desta et al., 2020). The structures of affitins and their partners were uploaded as “ligand” and “receptor”, respectively.

We evaluated the performance of the four available scoring schemes (“balanced”, “electrostatic-favored”, “hydrophobic-favored” and “van der Waals + electrostatics”) using as a measure of the capability of identifying the experimental assembly a parameter (crystal_RMSD from now on) describing the distance of the docking model from the crystallographic structure. crystal_RMSD was calculated by: 1) fitting the C_α_ atoms of the “receptor” (the bigger protein) of the docking models on the same atoms of the crystallographic structure; 2) calculating the RMSD of the C_α_ atoms of the “ligand” (the smaller protein) of the docking models with respect to the crystallographic structure. We considered as “Native” docking poses having crystal_RMSD ≤0.5 nm, while docking poses with crystal_RMSD >0.5 nm were considered as “Non-Native”.

The reranking of the first ten docking poses based on the crystal_RMSD parameter highlights that, for these complexes, the “balanced” scoring scheme performs better than the others (Supplementary Table S1), therefore we chose to use only this one for subsequent studies.

### Docking poses reranking by MD simulations

Among the methods available for the reranking of docking binding poses, the protocol proposed by Jandova et al. was applied. Briefly, it consists in performing MD simulations of the docking poses and monitoring parameters that describe their stability along the simulation time, thus allowing to discriminate among stable poses, i.e. probably Native, and not stable ones, probably Non-Native.

For each complex listed in Table 1, we carried out MD simulations of the crystal structure, of the two poses with the lowest crystal_RMSD values and of the two poses with the highest ones. We labeled the pose with the lowest crystal_RMSD value as **A**, and the one with the second lowest value as **B**. **C** and **D** are the labels for the poses having the second highest and the first highest crystal_RMSD values, respectively.

**TABLE 1:** Affitin-Protein complexes analysed.

PDB code	Affitin partners
4CJ0	endoglucanase D
4CJ1	endoglucanase D
4CJ2	lysozime C
5ZAU	tyrosine-protein kinase Fyn
6QBA	retinol-binding protein 4
5UFE	wild-type K-Ras(GNP)
5UFQ	K-RasG12D (GNP)

Complexes labelled by their Protein Data Bank (PDB) code. The partners that the affitins interact with are also indicated.

All MD simulations have been performed with Gromacs, release 2020.6 (Abraham et al., 2015) and visualized with Virtual Molecular Dynamics (Humphrey et al., 1996). The united atom Gromos 53A6 force field (Oostenbrink et al., 2004) has been used together with the SPC water model. Proteins have been centered in cubic or dodecahedral boxes, keeping a 1 nm minimum distance from the edges, and solvated with water molecules. Chloride and sodium ions have been added for maintaining the electroneutrality. Periodic Boundary Conditions (PBC) have been applied in the three dimensions. The systems have been minimized with steepest descent and conjugate gradient algorithms until a convergence criterium of 100 kJ mol^−1^ nm^−1^ was reached. Atoms motion equations have been integrated with leap-frog algorithm every 2 fs. A 1.4 nm cut-off was applied to van der Waals and electrostatics interactions, beyond whom the latter have been treated with PME (Darden et al., 1993). The set-up of equilibration and production runs followed reference (Jandova et al., 2021). After minimization, initial velocities have been generated from a Maxwell distribution at 50 K with a random seed. Then, systems have been progressively heated up (50, 150, 300 K) while the heavy atoms were positionally restrained with decreasing force constants (1000, 100, 10 kJ mol^−1^ nm^−2^). Production runs have been performed in NPT ensemble at 1 bar and 300 K. Proteins and solvent have been coupled to two velocity-rescaling thermostats (Berendsen et al., 1984; Bussi et al., 2007) every 0.1 ps and to a Berendsen barostat every 1 ps. All bonds have been constrained with LINCS (Hess et al., 1997). Analyses were performed every 500 ps. Two replicas (100 ns each) were carried out for the crystal structures and for each docking pose considered. The total simulation time for each complex sums up to 1 μs.

The CAPRI parameters (Méndez et al., 2003) interface-RMSD (i-RMSD), ligand-RMSD (L-RMSD), and the fraction of native contacts (Fnat) are widely used for the quantification of the quality of a docking model. They are defined and calculated for all simulated systems as follows: 1) i-RMSD. Interface residues are as those having at least one atom within 10 Å of an atom of the other protein. The RMSD of the backbone of these residues is then calculated during the MD simulation after the fitting on the backbone of the same residues in the reference structure, i.e. in the docking model; 2) L-RMSD. The RMSD of the backbone atoms (N,C_α_,C,O) of the ligand (the smaller of the two proteins) is calculated during the MD simulation after the superimposition of the same atoms of the receptor (the larger protein) on the reference structure; 3) Fnat. Pairs of residues on different sides of the interface were considered to be in contact if any of their atoms were within 5 Å. Fnat is calculated as the number of native (correct) residue–residue contacts during the MD simulation divided by the number of contacts in the reference structure. DockQ parameter (Basu and Wallner, 2016), describing the overall quality of the model, was calculated too. It ranges from 0 to 1: if DockQ ≥0.80 the model is a high quality one, if 0.80 > DockQ ≥0.49 the model quality is medium, acceptable if 0.49 > DockQ ≥0.23 and incorrect if DockQ <0.23. We also monitored the buried surface area (BSA), the number of hydrogen bonds (HB) and the protein-protein interaction energy (EPP).

Principal Component Analysis (PCA) was performed on the correlation matrix of Spearman coefficients of average values of the CAPRI parameters and of relative standard deviations (rSD) of BSA, HB and EPP. The analysis was conducted on the whole trajectories (production run).

### Docking poses reranking by MLCE

MLCE method was used for identifying areas on the partners of the affitins prone to an interaction (from now on, patches). This approach combines the analysis of a given protein’s energetic properties with that of its structural determinants, to identify protein areas that are prone to interact with potential partners.

MLCE is based on the hypothesis that some residues stabilize the protein folding, while others establish interactions with partners. The analysis of the interaction energy that each residue establishes with all other residues of the protein accounts for these different roles: residues which strongly interact with the rest of the protein are related to the stabilization of the folding core, while the recognition sites may have weaker pair interactions, as in this way they can easily undergo conformational changes which can make the protein able to recognize and bind a partner. More specifically, the analysis of the interaction energies of all the amino acids in a protein consists in calculating for each residue the non-bonded part of the potential energy (van der Waals, electrostatic interactions, solvent effects) *via* a MM/GBSA calculation. The resulting symmetric N×N interaction matrix $M_{ij}$ (where N is the number of residues of the protein) is then diagonalized and decomposed in eigenvalues and eigenvectors. The first eigenvector is then used to rebuild the energy matrix and multiplied with the contact matrix, which is built from the protein structure, through the Hadamard product, obtaining the MLCE matrix. This matrix is then used to rank spatially contiguous residue pairs with respect to the strengths of their energetic interactions (weakest to strongest). Potential interacting zones are then selected based on the spatial proximity of residues pairs showing the lowest energetic coupling with the rest of the protein, using a “weakness cutoff” of 15%, corresponding to the top 15% spatially contiguous residue pairs with the lowest-energy interactions. For an in-depth explanation of the method, refer to: Tiana et al., 2004; Morra and Colombo, 2008; Scarabelli et al., 2010; Genoni et al., 2012. MLCE has been extensively validated, also in experimental contexts. See for example: Peri et al., 2013; Gourlay et al., 2015; Sola et al., 2018; Marchetti et al., 2019; Bergamaschi et al., 2019; Serapian et al., 2020.

For the calculations, REBELOT program, version 1.3.2 (https://github.com/colombolab/MLCE), was used in cluster mode. Calculations were performed on centrotypes of clusters which cover at least 90% of the conformation variability sampled during three independent MD simulations (100 ns each). Patches were predicted on the centrotype of the most populated cluster considering the top 15% or top 10% of spatially contiguous residue pairs with the lowest-energy interactions. Then, they were compared with residues interacting with the affitins in the crystallographic structures and in the docking poses.

## Results

PDB codes of complexes of affitins and protein partners considered in the present work are listed in Table 1, together with the name of their partner. The chains selected for docking calculations are listed in Supplementary Table S2).

### Affitins—Partners docking

Docking between affitins and their partners was performed with the “balanced” scoring scheme in ClusPro (Kozakov et al., 2017; Vajda et al., 2017), as explained in the Methods section. For all the complexes considered, one or more Native poses among the first ten have been identified. In particular, for complexes 4CJ0, 4CJ1, 6QBA, and 5UFE, two Native poses are found (crystal_RMSD ≤0.5), while the pose with the second lowest crystal_RMSD value are Non-Native for complexes 4CJ2, 5ZAU and 5UFQ.

Calculation of the crystal_RMSD highlights that the ranking coming from the docking score function is often partially wrong, as shown in Table 2 which presents the reranking of the first ten docking poses (labelled in the docking server from 0 (best) to 9 (worst)) based on the crystal_RMSD values. These values are listed in Supplementary Table S1.

**TABLE 2:** Reranking of the first ten docking poses.

	4CJ0	4CJ1	4CJ2	5ZAU	6QBA	5UFE	5UFQ
Pose **A**	**0 (N)**	**0 (N)**	**0 (N)**	**0 (N)**	**2 (N)**	**0 (N)**	**0 (N)**
Pose **B**	**4 (N)**	**1 (N)**	**4 (NN)**	**3 (NN)**	**0 (N)**	**3 (N)**	**4 (NN)**
	9	2	3	2	1	7	8
	7	4	6	6	8	2	5
	2	5	2	1	9	5	7
	6	6	7	5	4	4	9
	3	3	9	7	3	1	1
	8	7	1	9	5	6	3
Pose **C**	**5 (NN)**	**9 (NN)**	**5 (NN)**	**4 (NN)**	**7 (NN)**	**8 (NN)**	**2 (NN)**
Pose **D**	**1 (NN)**	**8 (NN)**	**8 (NN)**	**8 (NN)**	**6 (NN)**	**9 (NN)**	**6 (NN)**

Docking poses are labelled as in ClusPro from 0 (best) to 9 (worst) based on the crystal_RMSD values (nm). The two closest (**A**, **B**) and the two furthest (**C**, **D**) poses from the crystallographic structure are shown in bold and labeled with N (Native), if crystal_RMSD ≤0.5 or NN (Non-Native) if crystal_RMSD >0.5.

Indeed, for all complexes, but 6QBA, the best docking pose found by the server is also the closest to the crystallographic structure; however, the ranking of the other poses does not correlate with their crystal_RMSD values. Hence, in a realistic case where the crystallographic structure of the complex is not available, a post-docking step aiming at evaluating the actual quality of the docking poses is necessary. Thus, we applied the two protocols described in the Methods section, in order to verify if they can be used to correctly identify the best models of the complexes.

### Docking poses reranking by MD simulations

MD simulations of complexes 4CJ0, 4CJ1, 4CJ2, 5ZAU, 6QBA, 5UFE and 5UFQ were carried out for the crystal structures and for the four docking poses **A**, **B**, **C**, and **D**, the first two poses being the ones closest to the crystallographic structures, whereas the second two are the poses that are furthest from their experimental counterparts (see also Methods section). Superimposition of the crystallographic structures and the docking poses are shown in Supplementary Figure S1.

The average values of three parameters i-RMSD, L-RMSD and Fnat were calculated from MD trajectories every 500 ps and the DockQ values have been evaluated (see Methods section). Table 3 reports the average values and standard deviations of DockQ from the two replicas of each system, obtained for the crystallographic structure of the complex and for poses **A** (Native for all the complexes), **B** (Native for all the complexes but 4CJ2, 5ZAU and 5UFQ), **C** and **D** (the latter two Non-Native).

**TABLE 3:** DockQ values and quality of the models.

PDB	Crystal	Pose A	Pose B	Pose C	Pose D
4CJ0	0.34 (0.07)—A	0.50 (0.03)—N/M	0.31 (0.04)—N/A	0.33 (0.04)—NN/A	0.39 (0.03)—NN/A
4CJ1	0.43 (0.07)—A	0.28 (0.03)—N/A	0.39 (0.03)—N/A	0.48 (0.04)—NN/A	0.23 (0.04)—NN/A
4CJ2	0.64 (0.03)—M	0.52 (0.04)—N/M	0.27 (0.03)—NN/A	0.19 (0.03)—NN/I	0.39 (0.07)—NN/A
5ZAU	0.39 (0.05)—A	0.33 (0.04)—N/A	0.27 (0.04)—NN/A	0.32 (0.03)—NN/A	0.34 (0.04)—NN/A
6QBA	0.48 (0.07)—A	0.44 (0.04)—N/A	0.30 (0.05)—N/A	0.34 (0.04)—NN/A	0.44 (0.04)—NN/A
5UFE	0.60 (0.04)—M	0.49 (0.04)—N/M	0.25 (0.05)—N/A	0.31 (0.04)—NN/A	0.29 (0.05)—NN/A
5UFQ	0.43 (0.09)—A	0.45 (0.03)—N/A	0.30 (0.04)—NN/A	0.20 (0.05)—NN/I	0.23 (0.03)—NN/A

DockQ average values (and standard deviations in parenthesis) derived from the two replicas of each system. Labels N or NN are used for denoting Native and Non-Native poses, respectively. Labels M, A, and I are used for indicating models of medium, acceptable, and incorrect quality respectively, on the basis of DockQ values.

As described in the Methods section, the quality of a model is defined as high/medium/acceptable/incorrect if the DockQ value is ≥0.80/≥ 0.49/≥ 0.23/< 0.23 (Basu and Wallner, 2016). It has to be remarked that none of the simulations performed on the crystallographic structure led to DockQ values equal or higher than 0.80, indicating a high quality of the model, and only two out of seven (4CJ2 and 5UFE) fall in the medium-quality area. For this reason, the discussion of the results obtained will not focus on the DockQ absolute values; rather, we will analyze the capability of DockQ parameter to correlate with the crystal_RMSD.

For what concerns poses **A**, three out of seven (4CJ0, 4CJ2 and 5UFE) are classified as medium quality ones. All of them are very close to the crystallographic structure of the complex (see crystal_RMSD values in Supplementary Table S1 and Supplementary Figure S1), so one could expect similar DockQ values between poses **A** and the corresponding crystallographic structures. Interestingly, in the case of 4CJ0, the value obtained for the crystallographic structure is lower, whereas it is slightly higher for the other two. For the other four complexes (4CJ1, 5ZAU, 6QBA and 5UFQ) DockQ values of the poses **A** fall in the acceptable quality range, with 4CJ1 being close to be classified as an incorrect model despite its optimal superimposition with the crystallographic structure (crystal_RMSD = 0.13). On the other hand, many DockQ values falling in the acceptable range were found for the poses **C** and **D**, even though they are Non-Native poses. The lowest DockQ values were obtained for Non-Native poses (**C** or **D**) only for three out of four complexes (pose **D** for 4CJ1, and **C** for 4CJ2 and 5UFQ). As for the other complexes, low DockQ values correspond to poses **B**, with some of them being good models (see crystal_RMSD values in Supplementary Table S1 and Supplementary Figure S1). In conclusion, DockQ can identify only four out of seven poses **A**.

Thus, we decided to analyze other structural and energetic parameters related to the protein-protein interface. In particular, we considered the buried surface area (BSA), the number of hydrogen bonds (HB) and the protein-protein interaction energy (EPP). Our focus was addressed to their relative Standard Deviations (rSD), rather than on average values, as the former directly reflects the docking pose changes during the MD simulations.

**FIGURE 2:**
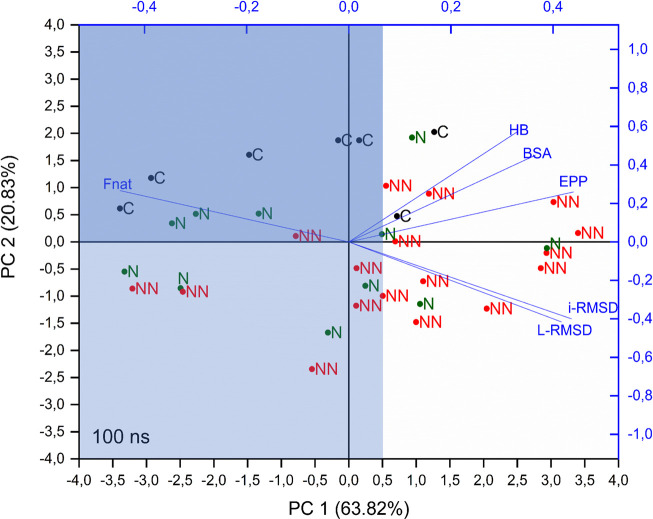
PCA biplot of considered parameters. PCA was performed on the correlation matrix of Spearman coefficients of CAPRI parameters (average values) and BSA, HB and EPP (rSD), calculated on the whole trajectory. C, N, and NN labels indicate crystallographic structures, Native and Non-Native poses respectively.

To get more insights on the capability of these parameters (or a linear combination of these parameters) in determining the quality of the models, Principal Component Analysis (PCA) was performed on the correlation matrix of Spearman coefficients of CAPRI parameters (average values) and of BSA, HB and EPP (rSD). The analysis was conducted on the parameters extracted from the whole trajectory (production run) and led to first two principal components (PCs) describing almost the 85% of the whole data set variability. The overlay of loadings and scores plots (biplot) obtained from the PCA is shown in Figure 2.

This analysis shows that i-RMSD, L-RMSD and Fnat are highly correlated, with Fnat pointing in the opposite direction: as expected, a high value of the latter is related to low values of i-RMSD and L-RMSD (see loadings of PC1 and PC2 in Supplementary Table S3). HB, BSA and EPP are highly correlated among each other and almost orthogonal to the CAPRI parameters, thus indicating the lack of direct relation with them. Concerning the components of each object (labeled in Figure 2 as C, N, NN for crystallographic structure, Native and Non-Native poses respectively), i.e. their position in the plane defined by PC1 and PC2, the analysis shows that: 1) PC1 is partially able to discriminate the poses: more than 70% of Cs and Ns fall at values of PC1 < 0.5 (light blue area in Figure 2), while 65% of NNs are above this value; 2) along PC2 a clear distinction is visible only for Cs, with all of them placed at PC2 > 0, while Ns and NNs are almost equally scattered. The intersection between areas defined by PC1 < 0.5 and PC2 > 0 is shown in dark blue in Figure 2.

Summing up, the PCA analysis can discriminate around 70% of Native and Non-Native poses, representing a useful tool to determine the quality of a docking pose in a realistic scenario, where the crystallographic structure of the complex is not available.

### Docking poses reranking by MLCE

MLCE was used for identifying patches on the partners of the affitins. It must be stressed that this method predicts protein area that can be recognized by a potential binding partner, meaning that in our case not all the predicted areas are regions which actually bind affitins. For the partner that binds the affitin in the complex 6QBA, a large patch involving all the interacting residues is found (Supplementary Figure S2A). For five out of the seven complexes analyzed (4CJ0, 4CJ1, 4CJ2 and 5ZAU), there is a partial overlay between patches predicted by MLCE and residues interacting with the affitins, as observed in the crystallographic structures (Supplementary Figure S2B), while the prediction for complexes 5UFE and 5UFQ does not include residues interacting with the affitins (Supplementary Figure S2C).

As in most the cases MLCE is able to identify the protein zone corresponding to the interacting residues, in principle it can be used as a tool for reranking docking poses. To this end, we decided to rank the poses based on the number of residues of the affitins interacting with residues belonging to patches identified on the other protein. In case the experimental structure is not available, all the patches should be considered.

As shown in Supplementary Table S4, for three out of the seven complexes (4CJ1, 5UFE, and 5UFQ), the highest number of affitins residues interacting with a patch is found for docking poses **C** or **D**, which are Non-Native. For complexes 5ZAU and 6QBA, this number is the same for two poses (**A** and **C** for the former, **A** and **B** for the latter). Only for complexes 4CJ0 and 4CJ2, affitins of pose **A** have the highest number of interacting residues with a patch. In other words, two (plus two doubtful cases) poses **A** can be identified based on MLCE analysis. These results indicates that a decision on the quality of a docking model cannot be based on this parameter only but, at the same time, it can be useful if coupled to another one, as shown in the following.

### Consensus approach DockQ—MLCE

In the present work, we used two different approaches to identify the correct binding mode of two interacting proteins, among the ones predicted through docking calculations. The first one is related to parameters that account for the stability of the binding pose, while the other is based on the prediction of the possible interacting residues of one of the two partners, through the analysis of the energetic and structural-dynamical properties of this protein.

In the sections above we showed that these methods, if applied individually, can lead to some misleading results, as they are not always able to identify the native binding mode among the predicted docking poses. Concerning DockQ, four poses **A** were identified as the best ones; in addition, for 6QBA, model **A** could not be distinguished from **D**. Considering instead MLCE results, only two poses **A** were identified, plus two doubtful cases in which poses **A** could not be distinguished from **B** or **C**.

However, since both methods show general good capabilities in discriminating the quality of the docking poses, and they rely on totally different assumptions, we apply a consensus approach which combine these two procedures into a single, more powerful method to identify the native conformation of complexes predicted through docking calculations.

In Table 4 we report, for each complex and each pose, the DockQ values and the number of residues of the affitins interacting with a patch.

**TABLE 4:** Selection of the models on the basis of DockQ and MLCE results.

Pose	Parameter	4CJ0	4CJ1	4CJ2	5ZAU	6QBA	5UFE	5UFQ
A	DockQ	**0.50**	**0.28**	**0.52**	**0.33**	**0.44**	0.49	0.45
	MLCE	**7**	**10**	**15**	**14**	**23**	1	0
B	DockQ	0.31	**0.39**	0.27	0.27	0.30	0.25	**0.30**
	MLCE	4	**3**	5	13	23	1	**1**
C	DockQ	0.33	0.48	0.19	0.32	0.34	**0.31**	0.20
	MLCE	3	0	9	14	18	**15**	2
D	DockQ	0.39	0.23	0.39	0.34	0.44	0.29	**0.23**
	MLCE	1	12	12	2	1	13	**9**
Selected model	DockQ	A	C	A	D	A/D	A	A
	MLCE	A	D	A	A/C	A/B	C	D
	DockQ + MLCE	**A**	**A/B**	**A**	**A**	**A**	**C**	**B/D**

**FIGURE 3:**
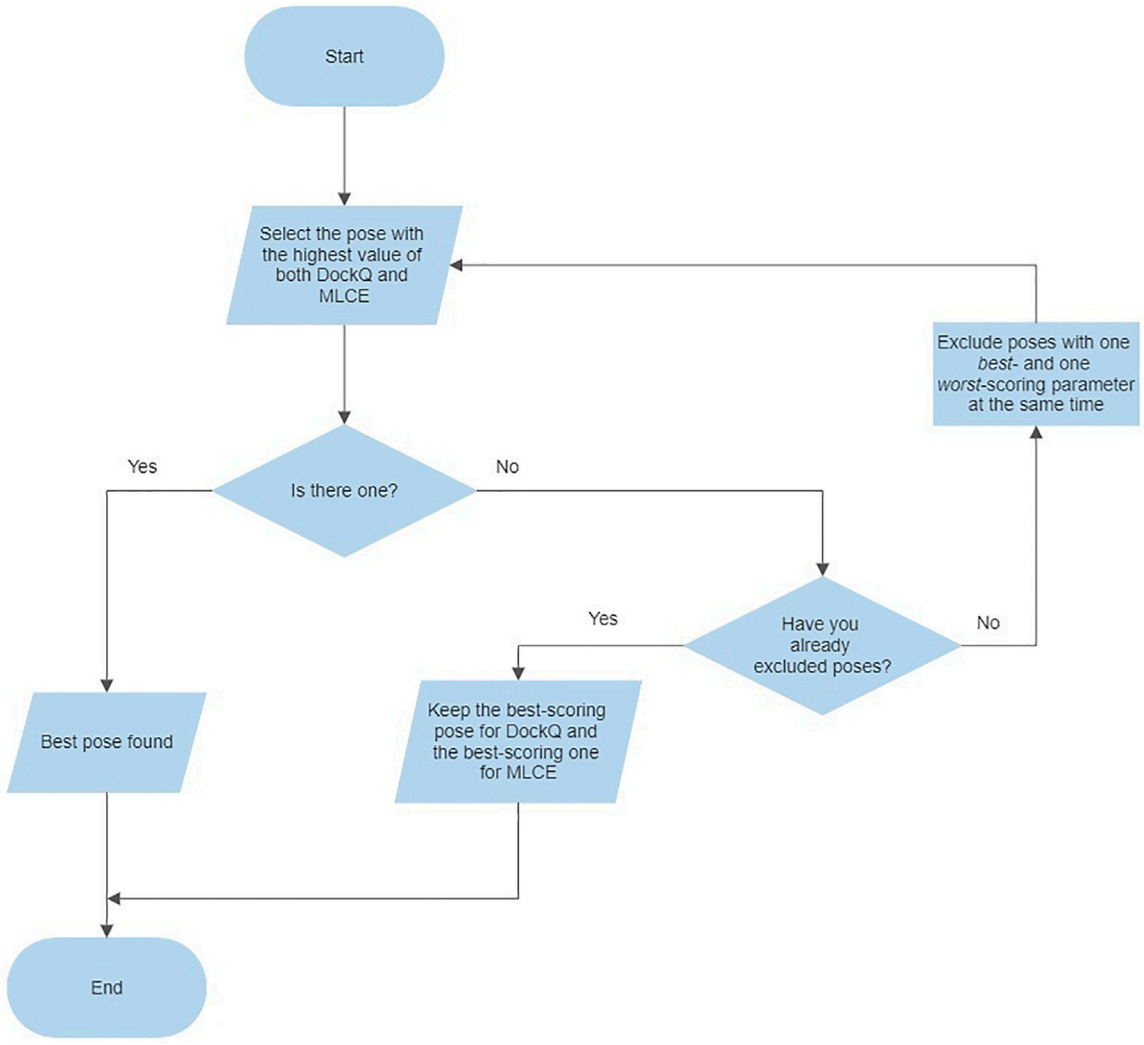
Flowchart describing the consensus approach protocol.

For each complex and each pose the DockQ values and the number of residues of the affitins interacting with a patch (labeled by MLCE) are reported. The selected models identified on the basis of the two approaches (alone or together, using the flowchart in Figure 3) are shown in bold.

For evaluating both parameters at the same time, we adopted the following criteria for each complex: 1) select the pose presenting the highest values of both parameters (4CJ0-**A** and 4CJ2-**A**); 2) if the previous point does not apply, exclude the poses characterized by one best- and one worst-scoring parameter at the same time (4CJ1-**C**, 4CJ1-**D**, 5ZAU-**D**, 6QBA-**D**, 5UFE-**A**, 5UFQ-**A**); 3) select the pose presenting the highest values of both parameters, i.e. repeat the first step (5ZAU-**A**, 6QBA-**A**, 5UFE-**C**); 4) if the previous point does not apply, select the poses presenting the highest value of each parameter, respectively (4CJ1-**A** and 4CJ1-**B**, 5UFQ-**B** and 5UFQ-**D**). Following this procedure, we identified unique poses in five cases: only for 4CJ1 and 5UFQ identification was not possible. This protocol is also described in a flowchart (Figure 3).

Considering that 4CJ1-**A** and 4CJ1-**B** are very similar, the combination of the two approaches allowed us to identify five correct models (four poses **A**, and one pose **A/B**), significantly improving the prediction obtained applying the two approaches individually.

## Discussion

In the present paper, we focused on the reliability of docking and post-docking procedures in predicting the binding geometries between affitins and several protein partners, by testing them on PDB-available complexes.

ClusPro proved to be a suitable docking approach for identifying putative binding geometries of complexes containing affitins. In fact, for all the seven complexes considered (4CJ0, 4CJ1, 4CJ2, 5ZAU, 6QBA, 5UFE, 5UFQ), at least one docking pose among the first ten well overlapping to the crystallographic structure was obtained, with crystal_RMSD values ranging from 0.13 to 0.28 nm. However, as it is known, the predicted binding modes obtained by the docking algorithm and the ranking, could be far from reality. Hence, we applied a method based on the monitoring, during MD simulations, of CAPRI parameters and the global parameter DockQ, in order to identify discriminants among Native and Non-Native poses. The idea at the basis of such an approach is that Native poses should be more stable during the MD simulations, i.e. the mutual arrangement of the two proteins should not change significantly. On the contrary, Non-Native models should undergo larger conformational changes, driving the two proteins away from the initial spatial arrangement. The results obtained showed that only three of the simulations performed on poses **A** (4CJ0, 4CJ2 and 5UFE) led to medium-quality (≥0.49) DockQ values. The remaining ones are acceptable, with two of them (4CJ1 and 5ZAU) characterized by values close to the incorrect range. Moreover, for some of the complexes (4CJ1, 6QBA), the obtained DockQ values for Native poses were sometimes too similar or even lower than the ones obtained for Non-Native poses. Summing up, DockQ was able to identify four out of seven poses **A**. Thus, we decided to look for other parameters, descriptive of the protein-protein interface, able to correctly rerank the poses. Among them, our attention was addressed to the number of hydrogen bonds between the two partners (HB), the buried surface area (BSA) and the protein-protein interaction energy (EPP). Principal component analysis (PCA) was performed on the correlation matrix of the Spearman coefficients of CAPRI parameters (average values) and of BSA, HB and EPP (rSD). The analysis was conducted on the whole trajectory (production run) and allowed us to observe that the first principal component is able to discriminate around 70% of Native and Non-Native poses. Concerning MLCE, the method showed to be an adequate tool for the prediction of the interacting residues in most cases. Thus, it was used for the reranking of docking poses, considering the number of residues of the affitins interacting with the patches identified on their partners. However, only two poses **A**, plus two doubtful cases, were identified. We thus decided to evaluate the quality of the docked poses by combining the results obtained through DockQ and MLCE methods, i.e. to employ a consensus approach. In this way, five out of seven correct models were detected.

## Conclusion

Affitins are small and highly stable proteins, features that make them easily engineerable. This results in them being ideal candidates for the development of molecular probes, entities that, through the specific recognition of a biomarker, are nowadays largely employed for the diagnosis of serious diseases such as tumor pathologies. Thanks to their structural stability, affitins-partners interactions can be studied by means of a simple protocol, as it is the one described in this paper. The protocol consists in the employment of ClusPro, a freely available server for protein-protein docking, which, treating the partners as rigid bodies, supplies the potential binding modes of the selected affitins and their partners.

The evaluation of the quality of the docking models is then addressed with two totally distinct approaches. The modeling of the full structural flexibility of the complex is introduced with MD simulations, in order to evaluate, at high atomic resolution, the structural details of the interface regions. Several parameters, including the CAPRI ones and the DockQ, are monitored and overall analyzed with PCA for quantifying the stability of docking models.

A totally different approach, the MLCE, is used in parallel to identify putative binding patches on the surfaces of the proteins. Docking models are reranked on the basis of the match with MLCE results.

DockQ and MLCE, if considered alone, are not fully able to discriminate among the docking poses. However, applying a consensus approach involving both the methods significantly improves the capability of identifying the correct binding mode among the predicted docking models.

Our work thus demonstrates that the combination of the MLCE method, which is completely unbiased, as it is based on the protein’s energetic properties and differs from the algorithm used by ClusPro to determine the potential binding surfaces, together with the dynamic evaluation of the stability of the predicted binding poses obtained with a rigid docking approach, is a fast and effective method to quickly discriminate correct from incorrect predictions generated by initial-stage docking and leads to better quality outcomes.

The overall approach we present here may represent an effective tool for evaluating a large number of affitins, and for selecting and designing new ones that are specific and selective for a target of interest.

We finally highlight that the presented protocol does not aim to be suitable for all protein-protein complexes. However, while applied to the case of affitins and known protein partners, no information about the characteristics of these specific complexes has been used to define it. In principle, the protocol can be transferable to other systems with similar characteristics, specifically, to those ones where no significant conformational rearrangement upon binding occurs.

## Data Availability

The raw data supporting the conclusion of this article will be made available by the authors, without undue reservation.
