# Supplementary material for: Evaluation of docking procedures reliability in affitins-partners interactions
Source: Front Chem. 2022 Dec 1;10:1074249. doi: 10.3389/fchem.2022.1074249 (PMC9752850; doi:10.3389/fchem.2022.1074249)
Supplement: Supplementary file 1 [file DataSheet1.pdf]

*Supplementary Material*

# Evaluation of docking procedures reliability in affitins-partners interactions

Anna Ranaudo[1*], Ugo Cosentino[1], Claudio Greco[1], Giorgio Moro[2], Alessandro Bonardi[1], Alessandro Maiocchi[3], Elisabetta Moroni[4]

[1]Department of Earth and Environmental Sciences, University of Milano-Bicocca, Milan, Italy
[2]Department of Biotechnology and Biosciences, University of Milano-Bicocca, Milan, Italy
[3]Bracco SpA, Milan, Italy
[4]Institute of Chemical Sciences and Technologies "G. Natta", National Research Council of Italy, SCITEC-CNR, Via Mario Bianco 9, Milan, 20131, Italy

*Correspondence: anna.ranaudo@unimib.it

**Supplementary Table S1.** Reranking of the first ten docking poses (labelled as in the docking server from best (0) to worst (9)) for the four scoring schemes on the basis of crystal_RMSD values (see paragraph 2.2). Crystal_RMSD values are reported only for the "balanced" scheme, that results the best performing scheme as for 6 out of 7 complexes the best docking pose found by the server is the closest to the crystallographic structure (lowest crystal_RMSD value). This number is 5, 4 and 1 for the "electrostatics", "hydrophobic" and " van der Waals + electrostatics" scoring scheme respectively.

**"balanced" scoring scheme**

| 4CJ0 | 4CJ1 | 4CJ2 | 5ZAU | 6QBA | 5UFE | 5UFQ |
|---|---|---|---|---|---|---|
| 0 (0.22) | 0 (0.13) | 0 (0.16) | 0 (0.28) | 2 (0.26) | 0 (0.20) | 0 (0.21) |
| 4 (0.30) | 1 (0.38) | 4 (0.72) | 3 (0.66) | 0 (0.33) | 3 (0.45) | 4 (0.55) |
| 9 (0.30) | 2 (0.41) | 3 (1.12) | 2 (0.89) | 1 (0.81) | 7 (0.52) | 8 (0.65) |
| 7 (0.40) | 4 (0.42) | 6 (1.20) | 6 (1.30) | 8 (0.94) | 2 (0.89) | 5 (0.69) |
| 2 (0.58) | 5 (0.54) | 2 (2.27) | 1 (1.46) | 9 (0.94) | 5 (1.19) | 7 (0.97) |
| 6 (0.58) | 6 (0.66) | 7 (2.45) | 5 (1.76) | 4 (0.97) | 4 (1.29) | 9 (0.99) |
| 3 (0.59) | 3 (0.71) | 9 (2.45) | 7 (1.86) | 3 (1.06) | 1 (1.40) | 1 (1.00) |
| 8 (0.77) | 7 (0.85) | 1 (2.51) | 9 (1.97) | 5 (1.13) | 6 (1.54) | 3 (1.12) |
| 5 (0.79) | 9 (0.98) | 5 (2.63) | 4 (2.03) | 7 (1.25) | 8 (2.56) | 2 (1.27) |
| 1 (0.80) | 8 (1.22) | 8 (2.73) | 8 (2.63) | 6 (1.94) | 9 (2.68) | 6 (1.73) |

**"electrostatics" scoring scheme**

| 4CJ0 | 4CJ1 | 4CJ2 | 5ZAU | 6QBA | 5UFE | 5UFQ |
|---|---|---|---|---|---|---|
| 0 | 0 | 0 | 0 | 1 | 2 | 0 |
| 5 | 7 | 8 | 2 | 0 | 0 | 9 |
| 3 | 3 | 9 | 5 | 2 | 7 | 8 |
| 6 | 2 | 1 | 3 | 7 | 3 | 3 |
| 2 | 4 | 5 | 1 | 3 | 6 | 6 |
| 4 | 5 | 7 | 6 | 8 | 4 | 1 |
| 9 | 1 | 2 | 9 | 4 | 8 | 4 |
| 1 | 9 | 3 | 7 | 5 | 1 | 5 |
| 7 | 6 | 6 | 8 | 6 | 5 | 2 |
| 8 | 8 | 4 | 4 | 9 | 9 | 7 |

**"hydrophobic" scoring scheme**

| 4CJ0 | 4CJ1 | 4CJ2 | 5ZAU | 6QBA | 5UFE | 5UFQ |
|---|---|---|---|---|---|---|
| 0 | 0 | 0 | 1 | 3 | 0 | 2 |
| 1 | 1 | 2 | 4 | 0 | 3 | 0 |
| 7 | 4 | 3 | 2 | 6 | 8 | 5 |
| 2 | 2 | 6 | 6 | 1 | 2 | 9 |
| 5 | 3 | 1 | 9 | 4 | 6 | 7 |
| 3 | 5 | 8 | 7 | 2 | 5 | 4 |
| 4 | | 4 | 3 | 5 | 1 | 1 |
| 6 | | 7 | 0 | 7 | 4 | 6 |
| 9 | | 5 | 5 | | 7 | 3 |
| 8 | | 9 | 8 | | 9 | 8 |

**"van der Waals + electrostatics" scoring scheme**

| 4CJ0 | 4CJ1 | 4CJ2 | 5ZAU | 6QBA | 5UFE | 5UFQ |
|---|---|---|---|---|---|---|
| 4 | 0 | 2 | 9 | 5 | 1 | 3 |
| 0 | 3 | 5 | 1 | 8 | 6 | 0 |
| 9 | 7 | 1 | 8 | 9 | 8 | 6 |
| 3 | 8 | 3 | 5 | 6 | 7 | 5 |
| 7 | 9 | 9 | 7 | 1 | 3 | 4 |
| 5 | 4 | 0 | 2 | 3 | 9 | 7 |
| 6 | 6 | 8 | 3 | 0 | 0 | 2 |
| 2 | 1 | 4 | 0 | 2 | 5 | 8 |
| 8 | 2 | 6 | 4 | 7 | 4 | 1 |
| 1 | 5 | 7 | 6 | 4 | 2 | 9 |

**Supplementary Table S2.** Proteins names and chains selected for docking calculations and following MD simulations. Where multiples chains were available, the one more structurally complete was chosen.

| P.D.B. code | Affitin: name*; chain | Affitin partner: name*; chain | Chains for docking calculations (affitin; partner) |
|---|---|---|---|
| 4CJ0 | E12 affitin; B | endoglucanase D; A (4CJ0) | B; A |
| 4CJ1 | H3 affitin; B | | B; A |
| 4CJ2 | affitin H4; D | lysozime C; B | C; B |
| 5ZAU | monobody binder; B | tyrosine-protein kinase Fyn; A | B; A |
| 6QBA | DNA-binding protein 7a; A | retinol-binding protein 4; A | B; A |
| 5UFE | R11.1.6; D (5UFQ) | wild-type K-Ras(GNP); A | B; A |
| 5UFQ | | K-RasG12D(GNP); A | C; A |

**Supplementary Figure S1.** Superimposition of the crystallographic structures and the docking poses. Affitins partners are shown in grey, affitins in the crystallographic structures in blue. Affitins in poses **A**, **B**, **C**, and **D** are shown in green, yellow, orange and red, respectively.

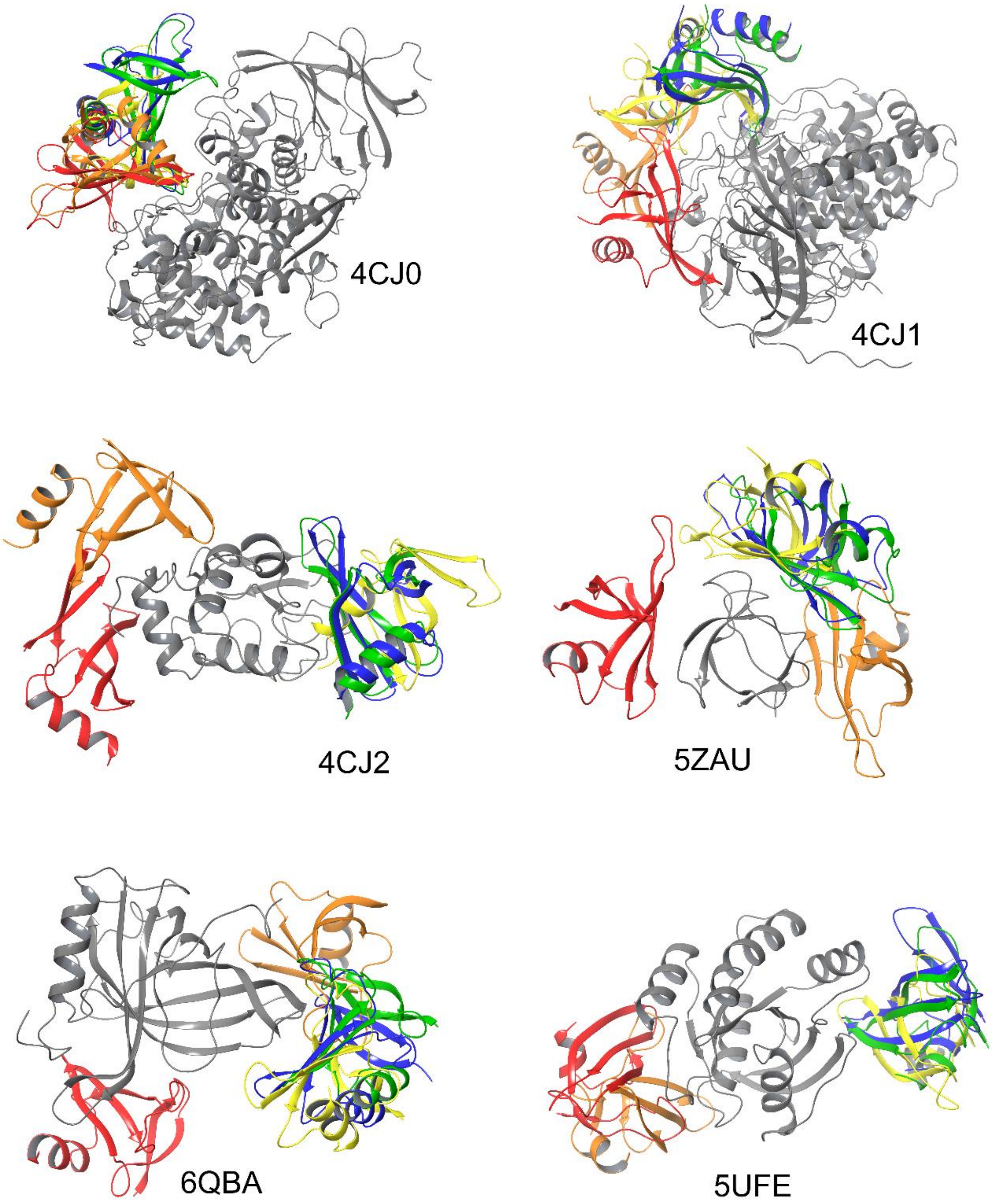

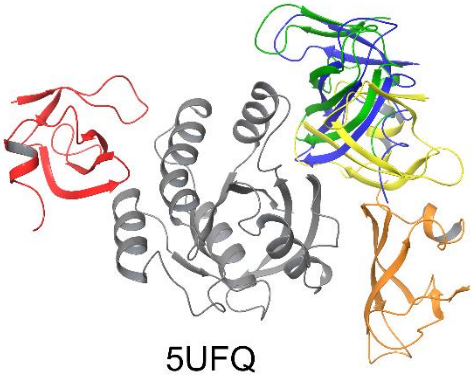


**Supplementary Table S3.** Loadings of principal components PC1 and PC2, obtained from the PCA performed as in paragraph 3.2.

| | PC1 | PC2 |
|---|---|---|
| L-RMSD | 0.4164 | -0.4157 |
| i-RMSD | 0.4362 | -0.3995 |
| Fnat | -0.4483 | 0.2665 |
| BSA | 0.3652 | 0.4504 |
| HB | 0.3294 | 0.5714 |
| EPP | 0.4399 | 0.2593 |

**Supplementary Figure S2A.** Complex 6QBA. Affitins in pink, partner in white. Patches calculated by MLCE are shown in blue, orange, green, red, brown, purple, deep teal, and dark green from the most energetically decoupled (patch 1) to the last one (patch 8), following the scheme:

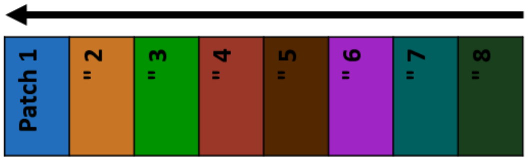


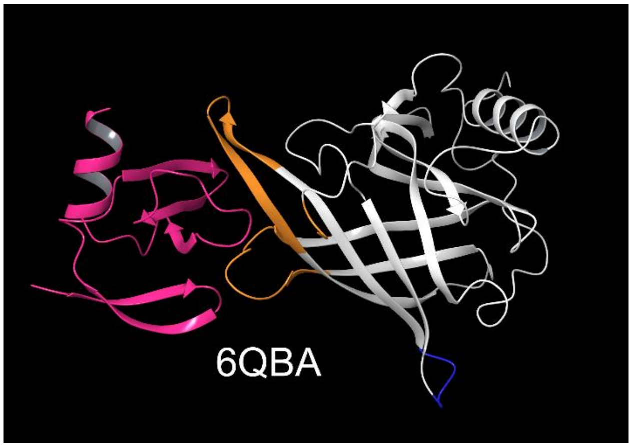

**Supplementary Figure S2B.** Complexes 4CJ0, 4CJ1, 4CJ2 and 5ZAU. Affitins in pink, partners in white. Patches calculated by MLCE are shown in blue, orange, green, red, brown, purple, deep teal, and dark green from the most energetically decoupled to the last one, following the scheme in **Supplementary Figure S2A.**

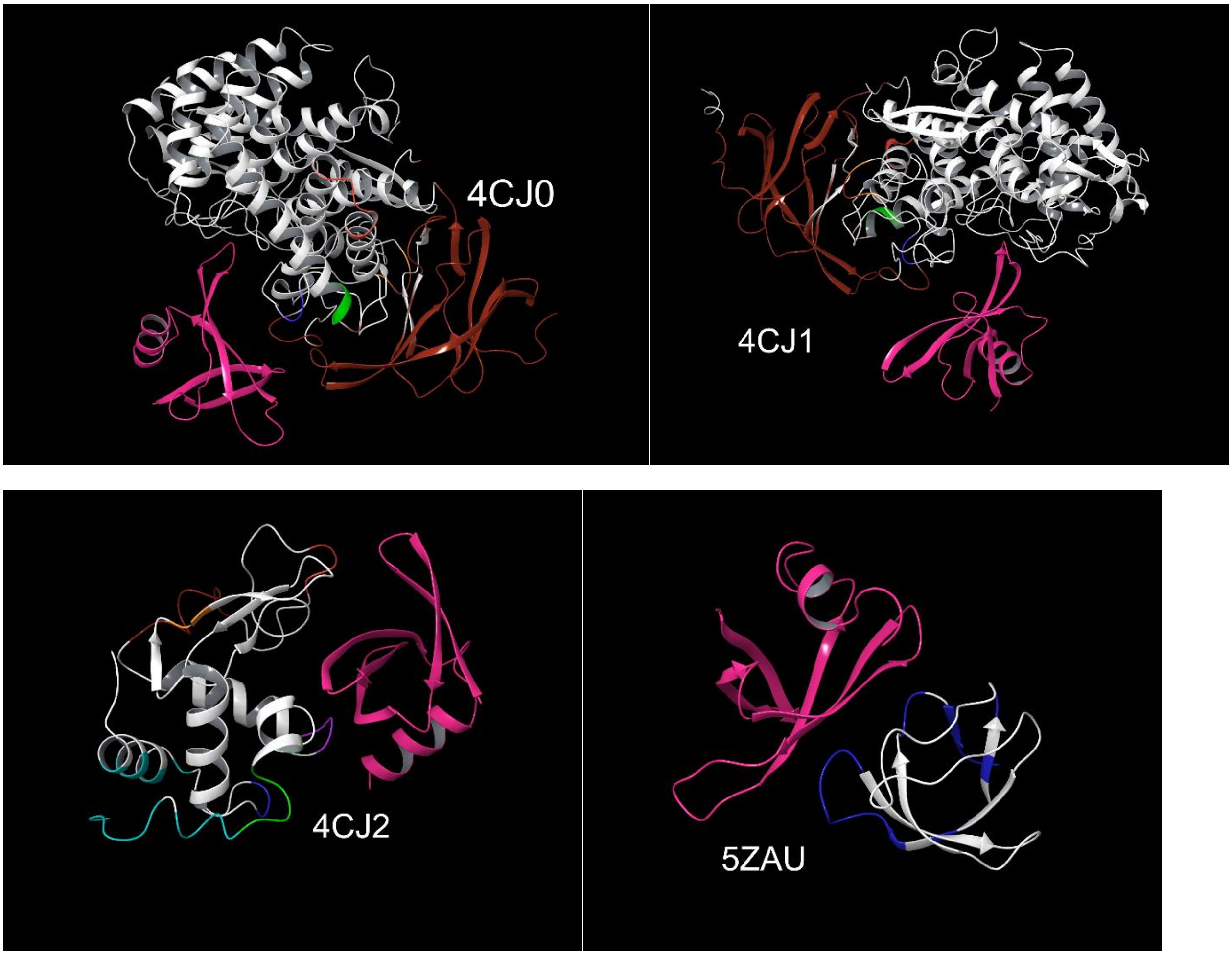

**Supplementary Figure S2C.** Complexes 5UFE and 5UFQ. Affitin in pink, partners in white. Patches calculated by MLCE are shown in blue, orange, green, red, brown, purple, deep teal, and dark green from the most energetically decoupled to the last one, following the scheme in **Supplementary Figure S2A.**

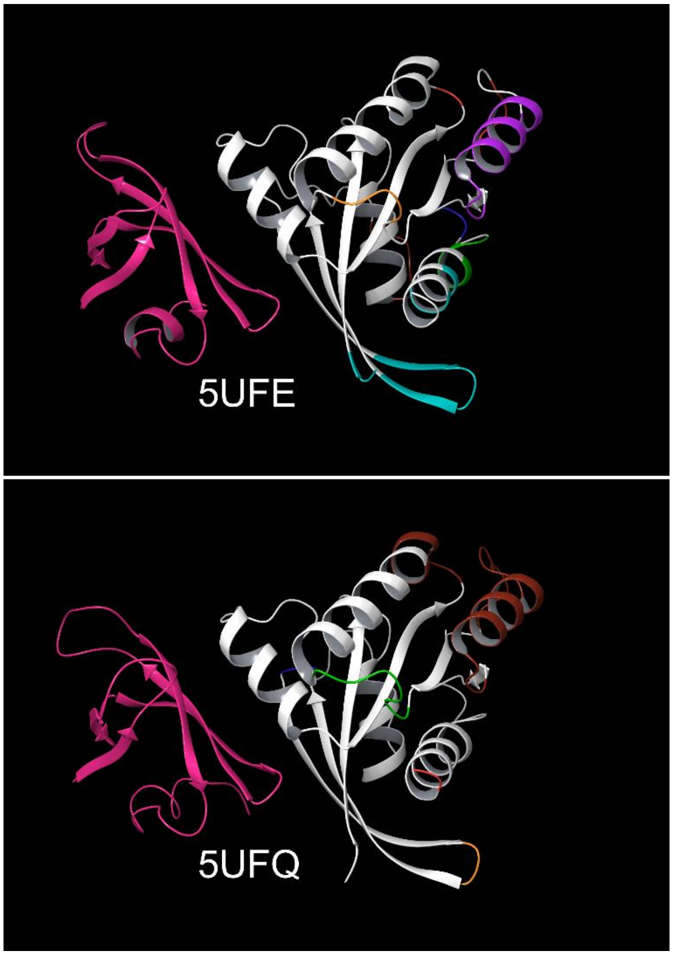


**Supplementary Table S4.** Number of residues of the affitins interacting with residues belonging to patches identified on the other protein, for the four docking poses and all the considered complexes.

| | 4CJ0 | 4CJ1 | 4CJ2 | 5ZAU | 6QBA | 5UFE | 5UFQ |
|---|---|---|---|---|---|---|---|
| **A** | 7 | 10 | 15 | 14 | 23 | 1 | 0 |
| **B** | 4 | 3 | 5 | 13 | 23 | 1 | 1 |
| **C** | 3 | 0 | 9 | 14 | 18 | 15 | 2 |
| **D** | 1 | 12 | 12 | 2 | 1 | 13 | 9 |
